# Interaction between Metformin, Folate and Vitamin B_12_ and the Potential Impact on Fetal Growth and Long-Term Metabolic Health in Diabetic Pregnancies

**DOI:** 10.3390/ijms22115759

**Published:** 2021-05-28

**Authors:** Manon D. Owen, Bernadette C. Baker, Eleanor M. Scott, Karen Forbes

**Affiliations:** 1 Discovery and Translational Science Department, Leeds Institute of Cardiovascular and Metabolic Medicine, University of Leeds, Leeds LS2 9JT, UK; ummdo@leeds.ac.uk; 2 Maternal and Fetal Health Research Centre, Division of Developmental Biology and Medicine, University of Manchester, Manchester M13 9WL, UK; bernadette.baker@manchester.ac.uk; 3 St Mary’s Hospital, Manchester University NHS Foundation Trust, Manchester Academic Health Science Centre, Manchester M13 9WL, UK; 4 Clinical and Population Sciences, Leeds Institute of Cardiovascular and Metabolic Medicine, University of Leeds, Leeds LS2 9JT, UK; E.M.Scott@leeds.ac.uk

**Keywords:** metformin, diabetes, placenta, folate, vitamin B_12_, one carbon metabolism, fetal growth, LGA, SGA, fetal programming

## Abstract

Metformin is the first-line treatment for many people with type 2 diabetes mellitus (T2DM) and gestational diabetes mellitus (GDM) to maintain glycaemic control. Recent evidence suggests metformin can cross the placenta during pregnancy, thereby exposing the fetus to high concentrations of metformin and potentially restricting placental and fetal growth. Offspring exposed to metformin during gestation are at increased risk of being born small for gestational age (SGA) and show signs of ‘catch up’ growth and obesity during childhood which increases their risk of future cardiometabolic diseases. The mechanisms by which metformin impacts on the fetal growth and long-term health of the offspring remain to be established. Metformin is associated with maternal vitamin B_12_ deficiency and antifolate like activity. Vitamin B_12_ and folate balance is vital for one carbon metabolism, which is essential for DNA methylation and purine/pyrimidine synthesis of nucleic acids. Folate:vitamin B_12_ imbalance induced by metformin may lead to genomic instability and aberrant gene expression, thus promoting fetal programming. Mitochondrial aerobic respiration may also be affected, thereby inhibiting placental and fetal growth, and suppressing mammalian target of rapamycin (mTOR) activity for cellular nutrient transport. Vitamin supplementation, before or during metformin treatment in pregnancy, could be a promising strategy to improve maternal vitamin B_12_ and folate levels and reduce the incidence of SGA births and childhood obesity. Heterogeneous diagnostic and screening criteria for GDM and the transient nature of nutrient biomarkers have led to inconsistencies in clinical study designs to investigate the effects of metformin on folate:vitamin B_12_ balance and child development. As rates of diabetes in pregnancy continue to escalate, more women are likely to be prescribed metformin; thus, it is of paramount importance to improve our understanding of metformin’s transgenerational effects to develop prophylactic strategies for the prevention of adverse fetal outcomes.

## 1. Introduction

Diabetes and its effect on fetal health are significant to the developmental origins of health and disease (DOHaD) hypothesis. Globally, around 223 million women currently live with diabetes, 60 million of whom are of reproductive age [1]. In addition to pre-existing diabetes, gestational diabetes mellitus (GDM), a form of maternal diabetes typically first diagnosed during weeks 24–28 of pregnancy, currently affects around 1 in 6 births worldwide, equating to approximately 16.8 million pregnancies [1,2,3]. The diagnostic criteria for GDM vary widely in different countries and in turn have led to heterogeneity in screening and trial designs, making it difficult for comparative judgement and unified consensus on its effect on maternal and fetal health [4]. If maternal hyperglycaemia is poorly controlled, this accelerates intrauterine growth and increases the risk of macrosomia, in which birth weight is > 4 kg, or the fetus being born large for gestational age (LGA), in which birth weight is above the 90th percentile. This may cause birth trauma for mother and baby by increasing the risk of preeclampsia, neonatal hypoglycaemia, shoulder dystocia, late stillbirth, or the need for caesarean section or neonatal intensive care [5,6,7]. Although GDM ceases post-parturition, these women are predisposed to an estimated sevenfold increased risk of type 2 diabetes mellitus (T2DM) within 5–10 years post-pregnancy [8,9,10]. Diabetes currently represents 10% of the National Health Service budget and, with the ever increasing prevalence of diabetic pregnancies, including diabetic risk factors such as the obesity epidemic and advanced maternal age [11,12], it is now paramount to refine diagnostic and treatment strategies to improve outcomes for mother and baby.

Insulin therapy is a standard treatment for diabetes to restore glucose homeostasis; however, this therapy is associated with increased maternal weight gain and hypoglycaemia. As rates of diabetes continue to rise, the cost, storage, and administration requirements for insulin have proven to be of increasing concern [13,14], particularly in developing countries where these storage requirements may not be feasible [14]. As such, metformin has been advanced as an alternative first-line therapy for T2DM and GDM in many countries [15,16].

## 2. Metformin in Pregnancy

**Figure 1:**
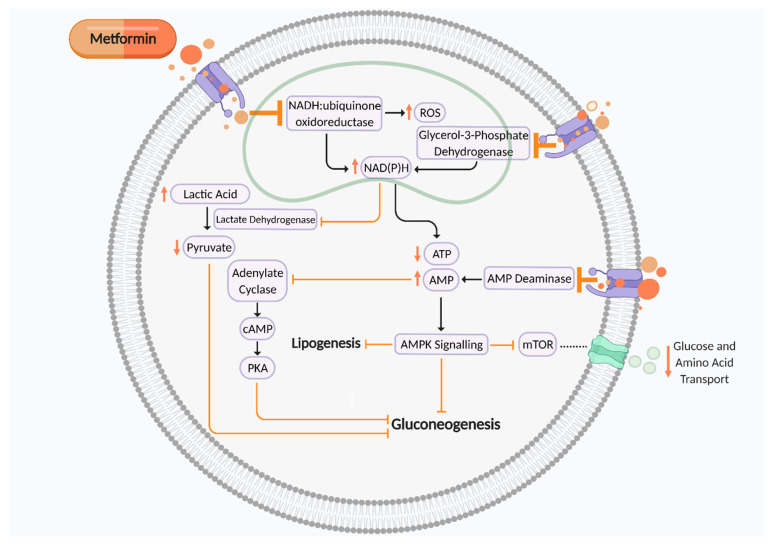
Putative mechanism of action of metformin on cellular metabolism and mitochondrial aerobic respiration to suppress gluconeogenesis. Metformin is an inhibitor of mitochondrial complex I (NADH:ubiquinone oxidoreductase), AMP deaminase, and mitochondrial glycerol 3 phosphate dehydrogenase (G3PDH), which all contribute towards suppression of cellular gluconeogenesis to maintain glycaemic control. ROS, reactive oxygen species; NAD(P)H, nicotinamide adenine dinucleotide phosphate; ATP, adenosine triphosphate; AMP, adenosine monophosphate; cAMP, cyclic AMP; PKA, protein kinase A; mTOR, mammalian target of rapamycin. Black arrows indicate cellular pathway. Orange arrows indicate putative effects of metformin. Figure created using Biorender.com.

Metformin is an oral synthetic guanidine analogue known as a ‘glucophage’ due to its glucose-lowering abilities by reducing gluconeogenesis and insulin resistance [15,16]. Metformin is a mitochondrial complex I (NADH:ubiquinone oxidoreductase) inhibitor which is transported into the cell to directly influence cellular respiration (Figure 1). Complex I (NADH:ubiquinone oxidoreductase) oxidises NADH synthesised from one carbon metabolism, glycolysis, fatty acid β-oxidation, and the tricarboxylic acid (TCA) cycle for adenosine triphosphate (ATP) production via the electron transport chain [17,18,19]. Thus, metformin-induced suppression of complex I increases NADH accumulation and ROS production and reduces ATP synthesis, thereby elevating the AMP:ATP ratio. This activates AMP-activated protein kinase (AMPK) and leads to inhibition of gluconeogenesis, therefore maintaining glycaemic control [17]. Metformin can also reduce gluconeogenesis by inhibiting AMP deaminase, which further contributes to elevated cellular AMP levels, thus in turn inhibiting adenylate cyclase and cAMP–PKA signalling. Metformin-induced suppression of mitochondrial glycerol 3 phosphate dehydrogenase (G3PDH) also augments cytosolic NAD(P)H concentration, leading to reduced pyruvate levels and a suppression of gluconeogenesis. However, activation of AMPK signalling inhibits mammalian target of rapamycin (mTOR) activity, a nutrient sensor which regulates amino acid transport and glucose storage [17,20,21,22,23] (Figure 1). This mechanism of action leads to improved insulin sensitivity by augmenting insulin receptor tyrosine kinase activity, amplifying glycogenesis and suppressing glycogenolysis, inhibiting lipolysis, enhancing glucose transporter GLUT4 recruitment and activity, and suppressing the activity of hepatic glucose 6 phosphatase. Metformin also heightens insulin release due to enhanced glucagon-like peptide-1 (GLP-1) activity [24].

Metformin’s glucose-lowering activity certainly demonstrates beneficial outcomes for maternal health, as it decreases maternal weight gain, inflammation, atherothrombosis, and cardiovascular disease mortality, all of which are diabetic co-morbidities [15,25]. However, studies suggest that its short- and long-term effects on the metabolic health of the offspring may not be as favourable. 

Whilst metformin therapy has been shown to significantly reduce the incidence of LGA, it has been reported that it may decrease birth weight to the extreme as metformin use in pregnancy is associated with an increased rate of small for gestational age (SGA) births; that is, those with a birth weight below the 10th percentile or two standard deviations below the mean weight for gestational age [5,7,26]. Notably, SGA offspring exposed to metformin in utero have shown signs of ‘catch-up growth’ during childhood. In the Metformin in Gestational Diabetes: The Offspring Follow Up (MiG: TOFU) study, at two years of age, metformin-exposed offspring demonstrated higher subcutaneous adiposity and larger mid-upper arm circumferences and bicep and subscapular skinfolds than insulin-exposed offspring [27]. By nine years of age, they presented with significantly higher body mass index (BMI) and larger arm and waist circumferences, triceps skinfolds, and abdominal fat volumes compared to insulin-exposed offspring [28]. A follow-up study of children exposed to metformin in utero in pregnancy complicated by polycystic ovarian syndrome also revealed they had higher BMIs at four years old than placebo-treated pregnancies [29]. Another randomised controlled trial showed that infants exposed to metformin during GDM pregnancy were markedly heavier at 12 and 18 months of age compared to insulin-exposed infants [30]. A murine study examining the effects of gestational metformin exposure from days E0.5 to E17.5 also showed that dams exposed to metformin manifested lower fetal weight on E18.5 than untreated dams. When fed a high-fat diet later in development, metformin-exposed fetuses were heavier than untreated fetuses and demonstrated increased mesenteric fat and liver weight. These findings, combined with gene set enrichment analysis of differentially expressed genes in the metformin and untreated murine offspring, reveal that metformin may induce transgenerational effects by way of fetal programming [31]. Accordingly, these studies suggest that metformin therapy in pregnancy may increase the risk of childhood obesity and thus is likely to predispose offspring to cardiometabolic diseases during adulthood. It is therefore crucial to develop our understanding of metformin’s mechanistic activity and its effects on the balance between maternal health and adverse fetal outcomes.

Studies from the literature around the effects of metformin treatment on the placenta suggest that metformin alters placental gene expression and function (Table 1), although the mechanisms remain unclear.

### 2.1. Transplacental Transport of Metformin

As the interface between maternal and fetal circulations, the placenta transports nutrients to the developing fetus. There is also evidence that metformin is transported across the placenta to the fetal circulation. In metformin-exposed pregnancies, serum samples from umbilical cord, placental, and fetal tissues have demonstrated metformin concentrations to be equal or greater than maternal levels, suggesting active transport of metformin from the maternal circulation across the placenta and into fetal tissue [44,45,46]. Metformin is a hydrophilic cation, has a half-life of 5 h, and is not metabolised in humans, but recent evidence suggests that metformin bioavailability, volume of distribution, and clearance may be significantly increased in pregnancy, dependent on dose [47,48]. The mechanisms of how pregnancy alters metformin clearance remain to be established. Although metformin can cross the placenta, it is undetermined how metformin influences placental metabolism to influence gene expression and whether fetal tissues handle metformin in the same way.

The transporter responsible for metformin uptake from the maternal circulation into the placenta is yet to be determined and requires further research. Studies have reported norepinephrine transporter (NET), serotonin transporter (SERT), and organic cation transporter novel type 2 (OCTN2) to be localised on the maternal interface of the placenta at the syncytiotrophoblast apical membrane, which could be responsible [19,49,50,51]. OCT3 has been demonstrated to be the key transporter for fetal metformin uptake and distribution, localised on the fetal interface of the placenta at the syncytiotrophoblast basal membrane and fetal capillaries. Indeed, OCT3-/- pregnant mice show attenuated fetal metformin exposure [19]. However, it is apparent that placental OCT3 expression increases with gestational age, as a murine study demonstrated that placental OCT3 mRNA and protein expression increased by 37-fold and 56-fold, respectively, at day 15 of gestation, and by 46-fold and 128-fold, respectively, at day 19 [52]. Thus, it is possible that metformin may not be reaching fetal tissues with significant concentration until late gestation. Moreover, these findings suggest that, unlike insulin [13], metformin can cross the placenta [21,28] and reach fetal tissue, which could potentially influence fetal growth and programming.

### 2.2. Impact of Metformin on Placental Nutrient Transport and Nutrient Bioavailabilty

It has been demonstrated that metformin influences fetal growth and nutrient bioavailability by inhibiting mitochondrial complex I, leading to activated AMPK signalling and inhibition of placental mTOR signalling (Figure 1). Attenuated placental mTOR signalling is associated with restricted fetal growth [53]. This mechanism of action may potentially explain the significant relationship between SGA births and metformin exposure in pregnancy. mTOR is highly expressed in the human placenta syncytiotrophoblast layer and mTOR complex 1 (mTORC1) signalling plays a major role in placental nutrient sensing, thus significantly influencing fetal nutrient availability and metabolism. Trophoblast mTORC1 regulates System A and System L amino acid transporters for amino acid uptake, essential for fetal metabolism [54,55]. Preliminary in vitro models of human trophoblast cells with silenced mTORC1 have also demonstrated that placental mTORC1 may regulate a circulating factor or factors, which could influence fetal growth [55,56]. mTORC1 signalling is regulated by placental insulin and IGF I, and fetal glucose, amino acid, and oxygen levels. Diabetes may increase mTORC1 activation due to elevated maternal nutrient and ATP concentrations [54,55]. During early gestation, the embryo mainly expresses immature mitochondria. As gestation develops, the placenta and fetus increase their expression of mature mitochondria, which are more susceptible to metformin inhibition. With this in mind, it is possible metformin may not adversely affect offspring growth until after the first trimester [21].

Transplacental metformin exposure may restrict placental and fetal growth by reducing nutrient bioavailability which could influence fetal programming. Evidence suggests metformin can influence the status of several vitamins and micronutrients, including vitamins B_1_, B_12_, and D, folic acid, and magnesium [15,20,57,58,59,60,61,62]. Whilst all of these nutrients are important for fetal growth and development, folate and vitamin B_12_ are co-factors of one carbon metabolism, essential for cell growth, metabolism, and production of the methyl donor S-adenosyl-methionine (SAM). Furthermore, exposure to metformin and maternal deficiency of both folate and vitamin B_12_, during pregnancy, lead to similar changes to placental and fetal growth and offspring health [63,64,65,66,67]. We therefore postulated that a potential mechanism by which metformin influences placental and fetal growth and offspring risk of cardiometabolic complications is by affecting the balance between folate and vitamin B_12_ levels and, therefore, perturbing one carbon metabolism. This review now focuses on this and discusses the evidence that supports this hypothesis.

## 3. One Carbon Metabolism

**Figure 2:**
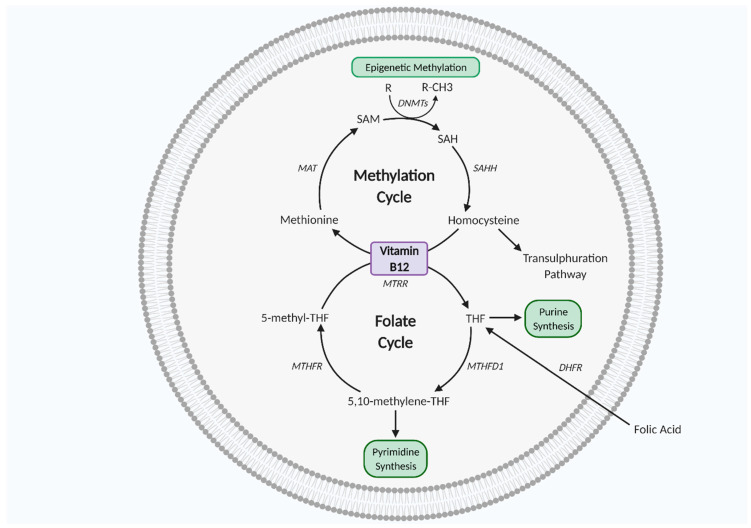
One carbon metabolism. Vitamin B_12_ and folate are co-factors of the methylation and folate cycles which interlink to complete the one carbon metabolism, essential for cell proliferation, differentiation, and growth. DHFR, dihydrofolate reductase; DNMTs, DNA methyltransferase; MAT, methionine adenosyltransferase; MTHFD1, methylenetetrahydrofolate dehydrogenase 1; MTHFR, methylenetetrahydrofolate reductase; MTRR, methyltransferase reductase; SAH, S adenosyl L homocysteine; SAHH, adenosylhomocysteinase; SAM, S-adenosyl methionine; THF, tetrahydrofolate. Figure created using Biorender.com.

Vitamin B_12_ and folate work synergistically as co-factors for one carbon metabolism, a biochemical network of methylating reactions and one carbon atom transfer vital for biosynthesis of DNA, RNA, lipids, amino acids, and neurotransmitters [63,68]. One carbon metabolism is also key for histone protein methylation to regulate gene expression and methionine and purine/pyrimidine synthesis to regulate cell growth, proliferation, and differentiation [63,68,69,70,71]. As such, one carbon metabolism is essential for in utero fetal development. One carbon metabolism is particularly important in mitochondrial redox homeostasis, as folate-mediated NADPH production plays a role in redox defence, thereby protective against oxidative stress [72]. In the cell cytoplasm, the folate cycle and methylation cycle work synergistically for purine/pyrimidine, methionine amino acid, and SAM synthesis. SAM is the primary DNA methyl donor synthesised from methionine via methionine adenosyl transferase (MAT) in the methylation cycle and DNA methylation is a crucial regulatory modification that regulates gene expression. SAM is converted to S adenosyl homocysteine (SAH) and then homocysteine (Hcy), which is then followed by regeneration of methionine, which completes the cycle. Hcy conversion to methionine requires synergism with the folate cycle, as 5 methyl tetrahydrofolate (5-methyl-THF) donates its methyl group to Hcy. The folate cycle intersects methionine synthesis via THF production from 5-methyl-THF. THF is then converted to 5-10-methyl-THF, followed by 5-methyl-THF regeneration via methylenetetrahydrofolate reductase (MTHFR) [63,70,71] (Figure 2).

Folate is found in the diet from fruit and vegetables and can be supplemented with its synthetic form, known as folic acid [68,69]. Folate deficiency elevates Hcy levels. High Hcy concentration is strongly associated with cardiovascular disease and may promote aberrant placental development, as in vitro trophoblast exposure to high Hcy leads to increased apoptosis and decreased human chorionic gonadotropin secretion [73,74]. Vitamin B_12_ is also a co-factor in one carbon metabolism [63,75]. Vitamin B_12_ is found in animal-derived foods and is essential for genomic stability and cellular metabolism, key for neurological and haematological developments, which have high cellular turnover rates [76]. Vitamin B_12_ deficiency inhibits methionine regeneration in one carbon metabolism and leads to Hcy and 5-methyl-THF accumulation [63,75] (Figure 2).

## 4. One Carbon Metabolism in Pregnancy

The one carbon metabolism cycle is critical for normal growth and development; hence, vitamin B_12_ and folate are essential micronutrients required for a successful pregnancy.

### 4.1. Vitamin B_12_

Heterogeneity exists in the reference ranges used by clinical studies for what is defined as ‘normal’, ‘low’, and ‘deficient’ levels of vitamin B_12_, as there is no gold standard criteria, thereby making direct comparisons between study results challenging [57]. However, most laboratories tend to use the following definitions of total serum B_12_ levels: normal (≥250 pmol/L), low (150–249 pmol/L), and acute deficiency (<149 pmol/L) [77].

Vitamin B_12_ deficiency has been associated with pernicious anaemia, neural tube defects, aberrant neurological development, insulin resistance, and, paradoxically, an increased risk of GDM [76,78,79,80,81,82]. Deficiency *in utero*, a crucial developmental window, increases the risk of intrauterine growth restriction and has been associated with increased risk of pre-term birth, a key driver of neonatal death and low birth weight or SGA [67,82,83]. This preventable risk is of particular concern in Asian women among whom SGA births are common, as diet is predominantly vegetarian and therefore low in vitamin B_12_ [84].

### 4.2. Folate

As defined by the World Health Organisation (WHO), folate deficiency is predicated on Hcy levels and marked as <10 nmol/L serum folate or <340nmol/L RBC folate [85]. Although plasma folate levels are highly influenced by transient diet and folate digestion and metabolism, RBC folate measurement is a good indicator of long-term folate status and has been shown to correlate well with tissue stores [69,86]. 

Low folate status is significantly associated with cognitive decline [87] and neural tube defects (NTDs) during gestation [88]. Low folate bioavailability is also associated with increased risk of spontaneous abortion and stillbirth [89]. A prospective study of UK pregnant adolescents has revealed that poor folate status is associated with increased prevalence of SGA births [66]; conversely, a positive relationship between RBC folate concentration and birth weight is supported by systematic reviews and meta-analyses in the literature [90,91]. Indeed, poor folate levels in pregnant teenagers have been associated with impaired placental trophoblast cell turnover and system A amino acid transport, demonstrating that low folate restricts placental growth and reduces nutrient transport to the fetus [65].

Low vitamin B_12_ and folate and perturbed one carbon metabolism have been associated with altered placental gene expression (Table 2) and thus may have a role in fetal programming [92,93,94,95,96]. The adverse impacts of vitamin B_12_ and folate deficiency on fetal development and birth weight and the similarities of these effects to in utero metformin exposure suggests there may be a link between metformin activity and alterations in the bioavailability or actions of these essential micronutrients.

## 5. Is Metformin Impacting on Fetal and Placental Development by Perturbing the One Carbon Metabolism Cycle?

### 5.1. Vitamin B_12_

It has been long reported that metformin may promote disturbances in vitamin B_12_ intestinal absorption [108]. Follow-up analysis shows metformin exposure promotes vitamin B_12_ malabsorption in 10–30% of people [58] and 30% of people may potentially experience deficiency [61,109]. Moreover, a meta-analysis review of 29 studies including a total of 8089 participants revealed people taking metformin were at significantly higher risk of developing vitamin B_12_ deficiency or insufficiency [110]. Women with GDM taking metformin have demonstrated low total vitamin B_12_ stores compared to those taking insulin [111]. Vitamin B_12_ insufficiency is associated with elevated Hcy levels, a phenomenon observed in women with polycystic ovarian syndrome who were taking metformin for 6 months, and also in people at risk of developing T2DM who were taking metformin for 10 years, thus demonstrating that metformin induces vitamin B_12_ deficiency at the tissue level [58,112]. 

The underlying pathophysiology of vitamin B_12_ malabsorption under metformin exposure has not yet been determined. Proposed theories suggest metformin promotes intestinal mobility disorders leading to bacterial overgrowth [113] and/or it may alter intrinsic factor secretion [114]. However, perhaps the most widely accepted theory is that metformin displaces calcium in the ileal surface membrane, leading to disruption in intestinal calcium-dependent vitamin B_12_ intrinsic factor uptake [115]. Theories on placental metformin vitamin B_12_ malabsorption are sparse. Several studies have demonstrated vitamin B_12_ receptors to be expressed in the placenta [116,117,118], which enter the endosomal lysosomal system upon ligand binding. Vitamin B_12_ is transported through the placenta and the fetal circulation by binding to transcobalamin (TC) proteins I, II, and III in blood [119]. TCs are usually produced in the liver; however, it has been shown that the placenta itself may produce TCs from early gestation [120,121], further demonstrating that vitamin B_12_ is metabolised by the placenta. As metformin therapy is associated with reduced holoTC levels [59], future studies investigating the effects of metformin on placental and fetal TC concentration may prove useful to determine whether metformin reduces placental and fetal vitamin B_12_ status by downregulating TC proteins. Pregnant rat models have also demonstrated that transplacental vitamin B_12_ transport to the fetus increases throughout gestation and placental vitamin B12 levels are consistently higher than maternal plasma and fetal tissue levels at every gestational stage [122]. This finding suggests that vitamin B_12_ may become increasingly important for fetal and placental growth throughout gestation and metformin-induced reductions in maternal vitamin B_12_ levels may have a more profound effect during late gestation.

It has been proposed that the placenta can adapt to low vitamin B_12_ status by upregulating angiogenesis-related genes to increase surface area for fetoplacental nutrient transport. This effect has exclusively been associated with female births. Indeed, placental endoglin (ENG) and vascular endothelial growth factor (VEGF) expression from female SGA and appropriate for gestational age (AGA) births were significantly negatively associated with maternal vitamin B_12_ status measured during the first trimester [97]. Vitamin B_12_ status may therefore induce fetal gender specific changes in placental gene expression which could potentially differentially impact fetal programming in male and female births. Controversy remains as to whether metformin promotes angiogenesis in all tissues [123,124,125,126]; however, evidence suggests that metformin may have pro-angiogenic effects in the placenta [37]. Future studies are needed to investigate whether metformin induces similar methylation patterns in placental angiogenesis-related genes as does low vitamin B_12_ concentration. By finding homologies between the pathogenesis of vitamin B_12_ deficiency and metformin, this will provide us with a better understanding of metformin’s mechanism of action in evoking adverse transgenerational effects on offspring through fetal programming.

Vitamin B_12_ deficiency in pregnancy has been associated with increased offspring and maternal metabolic risk due to altered lipid profile. Genome-wide and targeted DNA methylation analysis has shown that vitamin B_12_ deficiency in cultured human adipocytes leads to hypomethylation of the promoter regions of genes related to cholesterol biosynthesis, low density lipoprotein receptor (LDLR) and sterol regulatory element binding protein 1 (SREBF1), leading to an increase in their expression [127]. Adaikalakoteswari et al. 2017 have also reported that human preadipocyte cell lines exposed to insufficient concentrations of vitamin B_12_ promotes altered expression of 12 miRNAs associated with adipocyte function and differentiation. This finding was reflected in blood samples of pregnant women with low vitamin B_12_ status, thereby suggesting vitamin B_12_ deficiency may increase the risk of maternal insulin resistance and obesity [128]. Perhaps this phenomenon may be reflected in the developing offspring by way of fetal programming. Indeed, maternal vitamin B_12_ deficiency in Wistar rat models has shown offspring to have adipocyte dysfunction, increased adiposity, and altered lipid metabolism, where they display increased levels of total cholesterol, triglycerides, IL 6, TNF-α, and leptin and reduced levels of adiponectin and IL-1β [129]. Henderson et al. 2018 also showed murine offspring of vitamin B_12_-deficient mothers supplemented with folic acid have higher adiposity and reduced β cell mass and proliferation. These findings are also demonstrated in children exposed to vitamin B_12_ deficiency during gestation [70]. Whether metformin therapy induces similar changes in lipid profile, promoter methylation, and miRNA expression, and if these effects extend to the fetus, is currently unknown. Nonetheless, a recent study including 87 women found no significant associations between vitamin B_12_ measurements at second and third trimesters of pregnancy and insulin resistance, infant weight, and placental weight, respectively [130].

The dose of metformin given to pregnant women varies between 500 and 3000 mg/day [24]; thus, the duration and dose of treatment may significantly influence vitamin B_12_ status in metformin users (Table 3). As metformin can cross the placenta [21], offspring exposed to higher or longer cumulative doses of metformin could be at increased risk of developing vitamin B_12_ deficiency, which may lead to pre-term birth and SGA. Routine screening for vitamin B_12_ deficiency may therefore need to be considered in metformin-exposed pregnancies in women with T2DM and GDM. 

### 5.2. Folate

It has been demonstrated that metformin therapy reduces plasma and red blood cell (RBC) folate concentration and increases Hcy levels (Table 4). 

Notably, metabolomic fingerprinting of breast cancer cells has shown that metformin may have tumour suppressor effects by mimicking antifolate activity. Breast cancer cells exposed to metformin demonstrated accumulation of ‘trapped’ 5-formimino-tetrahydrofolate (THF), a folate metabolite and intermediate of one carbon metabolism. Folate-dependent target proteins were also inhibited under metformin exposure, leading to disturbed one carbon metabolism and reduced de novo purine/pyrimidine synthesis [62]. This suggests that metformin exposure in utero may impair fetal growth. However, a prospective cohort study of 336 pregnancies with first trimester metformin exposure revealed no increased risk of major birth defects and spontaneous abortions [134]. Regardless, screening of maternal folate should be considered as part of the therapeutic regimen for metformin-exposed pregnancies to elucidate any associations between placental development and fetal outcomes.

*In vitro* studies of folate deficient human cytotrophoblast cells show reduced mTOR signalling and amino acid transport, both of which are hallmarks of SGA placentas [103,104,135]. In vivo murine models have also demonstrated that maternal folate deficiency leads to lower fetal weight, reduced placental mTOR signalling and reduced systems A and L amino acid transporter activity [107]. Low folate bioavailability may therefore restrict fetal growth by reducing mTOR activity and placental nutrient transport. Immunohistological analysis has demonstrated reduced mTORC1 phosphorylation signalling in human intrauterine growth restriction (IUGR) placentas compared to appropriate for gestational age (AGA) placentas [136]. Reduced mTOR signalling and expression have also been observed in IUGR cytotrophoblast cells compared to normal term pregnancies [137,138]. Although these studies measured different mTOR phosphorylation targets for signalling activity analysis, both demonstrated downregulated mTOR signalling in association with IUGR. As IUGR- and metformin-exposed pregnancies are characterised by low folate status and similar outcomes as SGA births, these results suggest that metformin may promote aberrant mTOR signalling and nutrient sensing. mTORC1 signalling is regulated via methionine-induced activation from one carbon metabolism. A protein known as SAMTOR binds to SAM in response to methionine levels, which in turn regulates mTORC1 signalling [139]. Whether SAMTOR is affected by folate levels is unknown.

How metformin induces low folate status is yet to be established. Placental folate transport is mediated through folate receptor α (FRα/FOLR1), reduced folate carrier (RFC), and the proton-coupled folate transporter (PCFT/HCPI). FRα and PCFT have been found to be expressed on the syncytiotrophoblast microvillous plasma membrane (MVM) at both first trimester and term placenta. At first trimester, RFC is localised at the MVM and cytotrophoblast plasma membrane and, by term, is found on the MVM and basal plasma membranes of the syncytiotrophoblast [144]. The presence of these transporters in first trimester placenta, as well as FRα knockout shown to be embryonically lethal [145], demonstrate that folate transport is essential for fetal growth from early gestation. Folate transporter protein expression has been shown to be reduced in placentas of SGA offspring [146]. Thus, future studies investigating the interaction of metformin with folate transporters would prove interesting to explore whether metformin may be competing with folate or downregulating transporter expression.

## 6. Is Metformin Influencing Fetal Programming by Disturbing One Carbon Metabolism?

Maternal folate status has the capacity to influence fetal programming via placental gene expression, which could potentially lead to transgenerational epigenetic inheritance [147]. miRNA array analysis has revealed that folate-sensitive placental microRNAs (miRNAs), miR-222 3p, miR-141 3p, and miR-34b 5p, were downregulated with low maternal folate levels [65]. As metformin induces low maternal folate status, these findings suggest metformin may potentially be targeting the same miRNAs to reduce placental cell turnover and cause placental dysfunction, thereby altering fetal nutrient transport and predisposing offspring to increased disease susceptibility. Indeed, offspring with isolated NTDs, a condition strongly associated with low folate bioavailability, exhibit low placental weight, placental hypermaturity, and pathological oedema in terminal villi compared to offspring without congenital anomalies [148]. 

**Figure 3:**
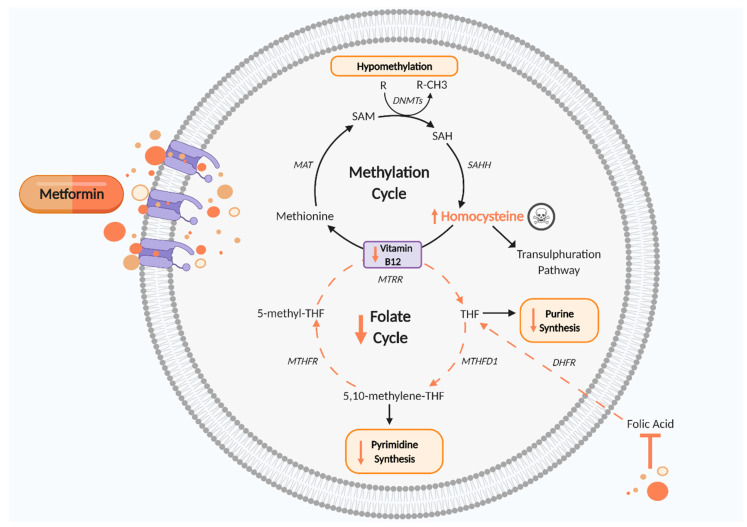
Disturbed one carbon metabolism induced by metformin. Metformin reduces vitamin B_12_, thus impairing the methylation cycle and leading to increased Hcy levels, which are cytotoxic, and hypomethylation of proteins and nucleic acids, which may cause epigenetic changes. The folate cycle is also disturbed by metformin’s antifolate-like activity, thereby reducing pyrimidine and purine synthesis and disrupting cell growth and proliferation. DHFR, dihydrofolate reductase; DNMTs, DNA methyltransferase; MAT, methionine adenosyltransferase; MTHFD1, methylenetetrahydrofolate dehydrogenase 1; MTHFR, methylenetetrahydrofolate reductase; MTRR, methyltransferase reductase; SAH, S adenosyl L homocysteine; SAHH, adenosylhomocysteinase; SAM, S-adenosyl methionine; THF, tetrahydrofolate. Black arrows indicate cellular pathway. Orange arrows indicate putative effects of metformin. Figure created using Biorender.com.

*In utero* development is a critical stage for cell differentiation and proliferation and requires a high turnover rate for growth. Limited bioavailability of methyl donors such as folate and vitamin B_12_ during this period may promote DNA hypomethylation and thus drive aberrant epigenetic and post-translational regulation by inducing differential gene and promoter methylation patterns. Post-translational methylation of N6-methyladenosine (m6A) on mRNA and primary miRNA transcripts plays a role in gene regulation and may also be altered with limited methyl donor bioavailability [149,150]. m6A methylation requires SAM as a methyl donor and therefore would be impacted by aberrant one carbon metabolism. Low maternal folate and vitamin B_12_ status in one carbon metabolism may promote genomic instability (Figure 3). Indeed, deficiency of both folate and vitamin B12 results in uracil retention and misincorporation in DNA synthesis rather than thymine, leading to aberrant base pair bonding with adenine, thereby inducing chromosome breaks [151]. Impaired DNA synthesis in utero may compromise placental and fetal development and increase the risk of fetal programming and disease susceptibility in offspring. This was observed by Zheng et al., who demonstrated that a reduction in mitochondrial DNA copy number was associated with insulin resistance in obese people [152]. 

Nicotinamide N-methyltransferase (NNMT) is an enzyme that ties one carbon metabolism with the methylation balance and nicotinamide adenine dinucleotide (NAD+) levels of the cell by catalysing nicotinamide (NAM) methylation via SAM. Elevated NNMT expression in murine white adipose tissue (WAT) has been associated with obesity and insulin resistance, whereas WAT NNMT knockout was protective [153,154]. It is already known that the dose of metformin may differentially affect cellular NAD+ levels [155]; however, whether metformin impacts NNMT expression requires further investigation.

Fetal programming induced by post-translational mechanisms from early life suboptimal nutrition has been demonstrated in adipose tissue of prediabetic adult rats and in human adults who were born SGA. It has been reported that, prior to manifestation of metabolic disease, miRNA-483-3p in adipose tissue is upregulated by suboptimal early life nutrition [156]. This miRNA is in intron 2 of the IGF2 gene and represses growth differentiation factor 3 (GDF3) expression, a protein from the transforming growth factor beta (TGFβ) superfamily that is key for lipid accumulation and adipocyte differentiation. Thus, downregulation of GDF3 may limit lipid storage and stimulate lipotoxicity and ectopic triglyceride storage, thereby promoting insulin resistance. Conservation of miRNA-483-3p upregulation in both SGA adult humans and rat suboptimal early life nutrition raises the question of whether this miRNA could be a biomarker for future metabolic syndrome risk [156]. Establishing whether metformin induces hypomethylation of IGF2, which leads to upregulated miRNA-483-3p and repressed GDF3, may give further insight into the possible mechanisms of metformin’s transgenerational programming capacity.

miRNA-483-3p upregulation has also been identified in maternal, placental, and fetal tissues of C57BL/6 mice deficient in both folate and vitamin B_12_ [105]. The same findings were true for miR-221 [105], an miRNA which has been shown to promote cardiovascular intimal thickening in diabetes [157]. Folate and vitamin B_12_ deficiency also led to downregulation of miR-133 exclusively in fetal tissues; however, this was tissue- and sex-specific [105]. Repression of miR-133 is a phenomenon associated with cardiomyopathy in diabetes; therefore, folate and vitamin B_12_ insufficiency in early life could be a risk factor for future disease susceptibility of the offspring. Global DNA methylation was reduced in maternal tissues with deficient levels of folate and vitamin B12 but was increased in fetal tissues. DNA methyltransferases DNMT1, DNMT3A, and DNMT3B were also increased in both maternal and fetal tissues. This strongly represents the significance of folate and vitamin B_12_ as methyl donors and their influence on gene regulation. Interestingly, RFC, PCFT, and FRα mRNA expression were upregulated in maternal, placenta, and fetal tissues with both folate and vitamin B_12_ insufficiency [105]. If metformin emulates these changes in epigenetic regulation, as shown by deficient folate and vitamin B_12_ levels, this will provide further evidence of the consequential downstream effects of metformin’s suppressive activity on methyl donors.

There is a considerable gap in the literature on the potential role of metformin in causing one carbon metabolism disturbance in the placenta and fetus and its impact on fetal programming. Future studies are needed to determine exactly how metformin exerts these disturbances, be it through directly decreasing vitamin B_12_ and/or folate levels, at a post translational level through miRNA regulation, or by directly targeting one carbon metabolism enzymatic action.

## 7. Vitamin Supplementation

Vitamin B_12_ and folate/folic acid supplementation may be an effective prophylactic approach to reduce the adverse effects of metformin on offspring. A cross-sectional study of people with diabetes taking metformin found that vitamin B_12_ deficiency was significantly decreased in those taking multivitamin supplements. As most multivitamin supplements include 6 to 25 µg of vitamin B_12_, this dose range may be sufficient for a protective effect for the fetus [60]. Another cross-sectional study of people with T2DM on metformin found multivitamin supplementation was associated with a marked decrease in vitamin B_12_ deficiency and lower risk of borderline deficiency [57]. The 1999–2006 National Health and Nutrition Examination Survey observed that a daily vitamin B_12_ dose over 0.6 µg reduced vitamin B_12_ deficiency and borderline deficiency by two-thirds in the general population, but this dose was not protective in people with T2DM who were taking metformin [61]. More clinical trials are needed to evaluate the protective dose of vitamin B_12_ supplementation in metformin users, particularly during pregnancy. Follow-up studies of offspring exposed to metformin during gestation and maternal vitamin B_12_ supplementation are also a priority to improve understanding of the long-term effects of vitamin supplementation on fetal programming. In a rat IUGR model, it has been reported that feeding the first generation (F1) offspring who have metabolic disease with a methyl donor-rich diet leads to prevention of metabolic disease being passed down to the F2 generation [158]. This suggests that postnatal dietary interventions may reverse the epigenetic effects of fetal programming in IUGR offspring and, therefore, postnatal strategies could also be applied to metformin-exposed pregnancies. Although not including people taking metformin, there are two on-going trials currently investigating the effects of vitamin B_12_ supplementation, pre-conceptually or during early pregnancy, on child development and the risk of diabetes [82,159]; the results of these studies have yet to be published but it will be important to monitor these to assess the potential beneficial effect of pregnancy supplementation. 

Folate or folic acid supplementation should also be considered as part of the therapeutic regimen for all metformin-exposed pregnancies. Currently, women with T2DM are advised to take pre-conceptual 5 mg/day of folic acid until 12 weeks gestation, which is more than the normally recommended peri-conceptual dose of 400 µg/day folic acid for non-diabetic women [160,161]. Policies of folate fortification in flour and cereal grain products have already been adopted in many countries to increase the peri-conceptual or early gestational folate exposure of the population. This has shown a reduction in the rate of NTDs and improvement in offspring cognitive function and bone mineral content and density [70,162,163]. This may be of particular importance in early-stage unplanned pregnancies. Indeed, high RBC folate concentration at 28 weeks gestation has been associated with higher birth weight [164], thus suggesting a possible clinical screening time frame and therapeutic window for intervention and maximum protection. However, as it is hard to monitor the intake of folate fortification in the general population, with some subjects also taking additional vitamin supplementation, there is a danger of excess consumption. This may promote adverse health outcomes, such as childhood insulin resistance, childhood asthma, aberrant child neurocognitive development, NK cytotoxicity suppression, and possible progression of premalignant and malignant lesions [162,163,165,166]. Pregnant mice supplemented with excess folic acid have demonstrated embryonic delay, embryonic growth retardation, and thinner embryonic ventricular walls [167]. Risks of folate/folic acid over-supplementation should therefore be considered when treating people with metformin. It has recently been reported that a high choline concentration in Wistar rat gestational diets may mitigate the negative effects of high folate levels on fetal programming in male offspring [168]. This finding may represent a potentially promising therapeutic avenue to be explored in humans who are at risk of folate/folic acid over-supplementation.

Balance and supplementation of both folate and vitamin B_12_ should be tightly controlled as studies have shown that high maternal folate and low maternal vitamin B_12_ levels are associated with offspring insulin resistance [163,169]. A murine study investigating the effects of maternal vitamin B_12_ deficiency and folic acid supplementation demonstrated that offspring from vitamin B_12_-deficient mothers showed glucose intolerance, fasting hyperglyceamia, and lower β cell mass. Offspring diet also influenced plasma insulin and fasting glucose levels [70]. These findings illustrate how offspring lifestyle and diet may influence disease susceptibility and could be additive ‘hits’ to the fetal reprogramming already induced by maternal stimuli. 

## 8. Future Considerations

Further research is needed to investigate the interaction between metformin and one carbon metabolism. Establishing if metformin directly targets one carbon metabolism enzymatic action or directly targets the cellular levels of both vitamin B_12_ and folate would provide insight into the causal relationship between metformin and its suppressive influence on one carbon metabolism. Deficient folate and vitamin B_12_ levels have been shown to promote epigenetic changes in gene methylation patterns and miRNA expression of placental and metabolic genes, which could lead to fetal programming. As such, future research would benefit from exploring the role of metformin in fetal programming by investigating its epigenetic effects in placental, umbilical cord, and fetal tissues. Establishing similarities in gene regulation, demonstrated by both deficient folate and vitamin B_12_ status, and metformin therapy may provide further insight into metformin’s mechanism of action, which would aid therapeutic innovation strategies. 

Emerging studies suggest that another potential mechanism by which metformin-induced impairment of one carbon metabolism may influence events in the placenta and fetus is via regulation of mitochondrial function. Yang et al. 2020 reported a new role for one carbon metabolism, in which it may be linked to mitochondrial respiration via NADH production of serine catabolism [18], whilst Boachie et al. 2021 demonstrated that B_12_ deficiency impaired mitochondrial respiration [170]. These findings, together with evidence of placental mitochondrial dysfunction in GDM pregnancy [171], suggest that further studies are needed to elucidate whether metformin-mediated B_12_/folate deficiency exerts a similar impact on mitochondrial function in diabetic pregnancy. However, one carbon metabolism is compartmentalised in the cell, where the cytosol and mitochondria have their own independent one carbon machinery. This may be important to consider when investigating the effects of metformin and whether it predominantly acts at mitochondria [172].

Studying the effects of metformin on placental and fetal growth in women with GDM or T2DM has its challenges. A lack of standardised diagnostic or screening criteria for GDM may leave many women undiagnosed and untreated, which could lead to inconsistencies in clinical trial design [173]. Pregnancy itself carries various contributing factors which may influence metformin activity and placental and fetal development, such as advanced age, weight, ethnicity, stage of diabetes, maternal glycaemic control, diet, lifestyle, pre-conceptual multivitamin intake, and sex of the offspring [174,175]. Metformin can alter the microbiome and lead to lactic acid accumulation, prompting further heterogeneity in vitamin B_12_ and folate absorption between individuals [15]. Better understanding of these variable factors will improve the efficiency of GDM clinical diagnosis and personalised therapeutic strategies. The dose and duration of metformin use should also be taken into consideration for patient screening regimens to facilitate personalised treatment.

As nutrient biomarkers are rapidly metabolised, this only gives us a transient snapshot of vitamin status and therefore makes it challenging to establish causal relationships of nutrient deficiency [176]. Heterogeneity in the diagnostic criteria and biomarkers for vitamin B_12_ deficiency promotes inconsistencies between clinical trial results [77], leading to a blurred diagnostic approach and ill-defined nutritional management policy. It is now suggested that other biomarkers, such as holotranscobalamin (active form of vitamin B_12_) and MMA, should be measured concurrently or as second line tests to validate true B_12_ deficiency, as serum vitamin B_12_ may not reflect accurate B_12_ levels in tissues or cells [176,177]. Heightened patient awareness of vitamin B_12_ and folate deficiency associated with metformin therapy would encourage patients to eat a more vitamin-rich diet, thus potentially decreasing the risk of adverse fetal health in metformin exposed pregnancy. 

## 9. Conclusions

**Figure 4:**
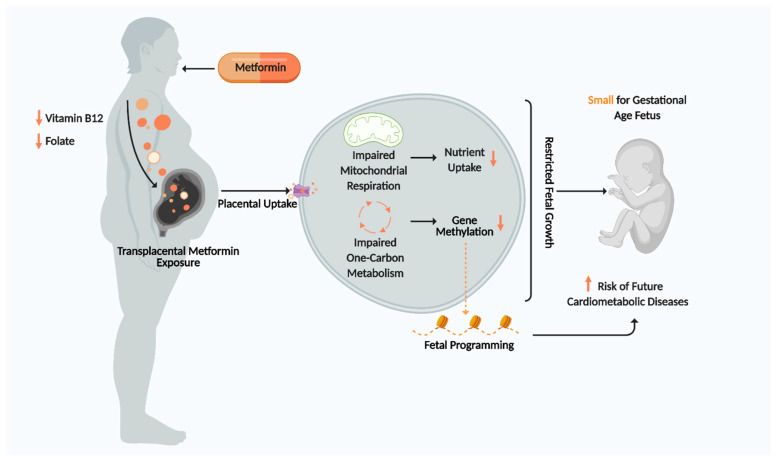
Summary of Proposed Mechanism for metformin effects on placenta and fetus. Figure created using Biorender.com.

Metformin is a first-line therapy for diabetes in many countries which vastly improves glycaemic control. However, its antifolate-like and vitamin B_12_-lowering activity may impose adverse transgenerational effects on offspring in pregnant T2DM and GDM women by impairing one carbon metabolism and mitochondrial aerobic respiration. This may restrict placental and fetal growth, thereby promoting SGA births and increasing offspring susceptibility to cardiometabolic diseases in adulthood (Figure 4). Currently, there are no gold standard criteria for GDM diagnosis and no routine tests to measure vitamin status of metformin users during pregnancy, leaving the fetus potentially vulnerable to harmful stimuli for a large part of gestation. Discovering an optimal therapeutic window for vitamin replenishment in metformin-exposed pregnancies may potentially improve fetal health and disease susceptibility. It is paramount that future clinical trials studying the effects of metformin on fetal outcome should have analogous designs and methodologies to allow for more comparable end points and improved data comparison and interpretation. Longer follow-up studies of offspring exposed to metformin are needed to evaluate the long-term effects of metformin on fetal programming and cardiometabolic health. Ultimately, discovering how metformin promotes SGA births and fetal cardiometabolic disease will enable us to design a novel drug that continues to exert the beneficial effects metformin has on maternal health whilst minimising adverse effects on fetal health.

## Figures and Tables

**Table 1:** Current literature on the impact of metformin on placental gene expression and function [32,33,34,35,36,37,38,39,40,41,42,43].

Reference	Model	Effects Demonstrated by Metformin	Significance
**Clinical studies**			
Jamal et al. 2012 [32]	Pregnant women with PCOS treated with metformin	- ⇔ on birth weight - ↓ uterine artery pulsatility index	Metformin adversely affected uteroplacental circulation
**Ex vivo or in vitro human placental studies**			
Jiang et al. 2020 [33]	Human GDM and T2DM placental explants cultured and treated with metformin *(ex vivo)*	Male human placental explants: - AMPK activation - ↑ H3K27 acetylation - ↓ DNMT1 protein abundance - ↓ PGC-1α promoter methylation and ↑ PGC-1α mRNA expression	Effects of metformin may be fetal sex-dependent Metformin may improve placental efficiency by facilitating placental mitochondrial biogenesis
Brownfoot et al. 2020 [34] Cluver et al. 2019 [35] Kaitu’u-Lino 2018 [36] Brownfoot et al. 2016 [37]	Human primary tissues exposed to metformin; placental explants, endothelial cells and placental villous explants, whole maternal vessels, maternal omental vessel explants *(in vitro* and *ex vivo)*	- ↓ sFlt-1 and sEng secretion from primary endothelial cells, preterm preeclamptic placental villous explants and villous cytotrophoblast cells - ↓ VCAM-1 mRNA expression in endothelial cells - ↑ whole maternal blood vessel angiogenesis - ↓ sFlt mRNA expression - ↓ TNFα-mediated endothelial cell dysfunction	Metformin enhances placental angiogenesis and reduces endothelial dysfunction by decreasing endothelial and trophoblastic antiangiogenic factor secretion via mitochondrial electron transport chain inhibition Metformin is being trialled as a medication for preeclampsia (trial number PACTR201608001752102)
Szukiewicz et al. 2018 [38]	Human placental lobules perfused with metformin under normoglycemic or hyperglycaemic conditions *(ex vivo)*	- ↓ CX3CL1 and TNFα secretion - ↑ placental CX3CR1 protein expression - ↓ placental NFκB p65 protein	Metformin has anti-inflammatory effects in the placenta
Correia-Branco et al. 2018 [39]	HTR-8/SVneo extravillous trophoblast cell line exposed to metformin *(in vitro)*	- ↓ proliferation - ↑ apoptosis - Inhibited folic acid uptake - Inhibited glucose uptake - Effects of metformin were prevented by inhibition of mTOR, JNK, and PI3K pathways	Metformin impairs placental development and nutrient transport via PI3K, mTOR, JNK, and PI3K pathways
Arshad et al. 2016 [40]	Human placental explants; from healthy pregnancy, non-treated diet-controlled GDM pregnancy, and metformin-treated GDM pregnancy *(ex vivo)*	- ↓ similar morphology in metformin-treated GDM placenta and non-treated healthy placenta, except for increased cord width - ↓ placental width in metformin-treated GDM placenta compared to non-treated GDM placenta - ↓ chorangiosis, placental thickness, and syncytial knots in metformin-treated placenta compared to non-treated GDM placenta	Metformin may improve placental morphology by restoring diabetic placental hallmarks to characteristics similar to healthy placenta
Han et al. 2015 [41]	Human first trimester trophoblasts treated with or without metformin *(in vitro)*	- ↓ trophoblast cytokine and chemokine release in normal and high glucose culture concentrations - No antiangiogenic or antimigratory effects	Metformin may potentially decrease placental glucose-induced inflammatory response
**In vivo rodent studies**			
Jiang et al. 2020 [33]	Mice treated with maternal metformin and high-fat diet	Improved placental efficiency in males: - ↓ PGC-1α promoter methylation and ↑ PGC-1α expression - ↑ TFAM expression Improved glucose homeostasis in male offspring	Metformin may improve placental efficiency by facilitating placental mitochondrial biogenesis Metformin may be protective to the offspring by suppressing epigenetic changes evoked by maternal diabetes
Wang et al. 2019 [42]	Pregnant mice fed an isocaloric diet (control), high-fat diet, or high-fat diet plus metformin *(in vivo)*	- ↓ placental weight compared to control - Partially rescued high-fat diet induced ↓ in placental and fetal weight - ↑ VEGF and MMP-2 protein expression	Metformin improves high fat diet-induced reduction in placental and fetal growth, potentially by modulating placental vasculature
Alzamendi et al. 2012 [43]	Pregnant rats fed a normal or high-fructose diet, treated with metformin *(in vivo)*	- ↓ fetal weight - ⇔ on placental weight or blood vessel area - Improved fructose diet induced ↓ blood vessel area	Metformin reduces fetal weight in mice fed a normal diet Metformin prevents high fructose diet-induced placental dysfunction

Dark grey is table heading; pale grey titles demonstrate whether the study was clinical, ex-vivo or in vitro human placental, or in-vivo rodent studies. ⇔ no change; ↓reduction; ↑ increase. AMPK, AMP-activated protein kinase; DNMT, DNA methyltransferase; PGC-1α, peroxisome proliferator-activated receptor-gamma coactivator 1α; TFAM, mitochondrial transcription factor A; sFlt-1, soluble fms-like tyrosine kinase-1; sEng, soluble endoglin; VCAM-1, vascular cell adhesion molecule 1; TNFα, tumour necrosis factor alpha; VEGF, vascular endothelial growth factor; MMP-2, matrix metalloproteinase-2; NF-κB, nuclear factor kappa B; mTOR, mammalian target of rapamycin; JNK, c-Jun N-terminal kinase; PI3K, hosphatidylinositol-3-kinase.

**Table 2:** Effects of folate and vitamin B_12_ status on the placenta [65,73,97,98,99,100,101,102,103,104,105,106,107].

Reference	Model	Functional Effects/Findings	Significance
**Clinical studies**			
Mani et al. 2020 [97]	Maternal first trimester B_12_ status correlated with term placental angiogenesis genes	*Vitamin B_12_ deficiency:* ↑ placental ENG and VEGF expression in female births only	Suggests placental adaptation to low maternal B_12_ by upregulating angiogenic pathways in a sex-specific manner
Baker et al. 2017 [65]	Prospective study of folate-deficient pregnant women	*Folate deficiency:* - ↑ trophoblast proliferation and apoptosis - ↓ amino acid transport - ↓ placental hormones (PAPPA, progesterone, and hPL) - ↑ placental miR-222-3p, miR-141-3p, and miR-34b-5p - ↓ ZEB2, MYC, and CDK6 mRNA expression in placenta	Folate deficiency adversely impacts on placental development and function and this may be via regulation of miRNAs in the placenta
**Ex vivo or in vitro human placental studies**			
Moussa et al. 2015 [98]	JEG3 cells exposed to 2nM (low), 20 nM (normal), or 100nM (excess) levels of folic acid	*Low folic acid: * - ↓ proliferation - ↓ cell invasion - ↓ cell viability *Excess folic acid: * - ↑ proliferation	Folate deficiency adversely impacts on placental development but excess folate may increase placental growth
Shah et al. 2016 [99]	BeWo and JEG cells exposed to 20ng/mL (normal) or 2000ng/mL (supraphysiological) folic acid	*Supraphysiological folic acid* - ↓ cell viability - ↓bhCG secretion (only in JEGs) - ↑↓EGFR mRNA - ↑oxidative stress - ↑TNF-a mRNA	Excess folic acid treatment has an adverse impact on placental growth, development, and function.
Yin et al. 2019 [100] Carletti et al. 2018 [101] Ahmed et al. 2016 [102]	HTR-8/SVneo, BeWo cell lines exposed to supraphysiological (2000ng/mL) or low (2ng/mL) levels of folic acid for 48hr	*Supraphysiological folic acid:* - ↓ cell viability in BeWo - ↑ proliferation rate in HTR-8/SVneo - ⇔ on apoptosis or β-hCG release - ↑ *tert*-butylhydroperoxide (TBH)-induced oxidative stress *Low folic acide:* - ↓ cell viability - ↓ cell invasion - ↑ autophagy - ↓ apoptosis - ↓ invasiveness	Both low and high levels of folate adversely impact on placental development
Rosario et al. 2017 [103] Di Simone et al. 2004 [73] Steegers-Theunissen et al. 2000 [104]	Primary trophoblast (third trimester) exposed to low folic acid	*Low folic acid:* - ↑ apoptosis - ↓ hCG secretion - ↓ mTOR signalling - ↓ activity of key amino acid transporters	Low folate impacts on trophoblast viability and may alter transport of nutrients to fetus
Ahmed et al. 2016 [102] Yin et al. 2019 [100]	Human villous explants (third trimester) exposed to supraphysiological (2000ng/mL) or deficient (2ng/mL) levels of folic acid for 48 hours	*Supraphysiological folic acid:* - ⇔ in any assessed functions *Low folic acid:* ⇔ in any functional assessments (Ahmed et al. 2016) [102] - ↑ apoptosis and autophagy (Yin et al. 2019) [100]	Limited effect observed in human placental explants suggests this may not be the optimal model for studying high/low folate
**In vivo rodent studies**			
Mahajan et al. 2019 [105]	Mouse dietary model—effect of the altered dietary ratio of folate and B_12_ on the expression of transporters, related miRNAs, and DNA methylation in maternal/fetal tissues in F1 and F2 generations	*Folate deficiency; folate over-supplementation; vitamin B_12_ deficiency; vitamin B_12_ over-supplementation; combination of folate/B_12_ deficiency/over-supplementation:* - Altered placental mRNA for folate transporters, B_12_ transporters/proteins, DNMT1, DNMT3A, and DNMT3B - Altered placental miR-483, miR-221, and miR-133 expression - Placenta global DNA methylation affected	Demonstrates that altered dietary ratios of folate and B_12_ can have more severe effects than the individual deficiencies
Shah et al. 2017 [106]	Rat dietary model fed normal (400 µg/day) or high (5 mg/day) folate +/- B_12_ (various forms)	*High folate:* - ↓ placental weight - ↓ offspring birth weight - ↓ miR-16 and 21 expression - ↑ plasma homocysteine *High folate combined with Vitamin B_12_ supplementation:* - Restored miR-16 and miR-21 expression - Prevented ↓ offspring birth weight	High folate reduces placental and fetal growth, potentially via altering miRNA levels in placenta. This is restored by vitamin B_12_ supplementation
Yin et al. 2019 [100]	Mice on folate-deficient diet	*Folate deficiency:* - ↓ placental size - ↓ endocrine function - ↓ placental vascularisation - ↓ trophoblast differentiation - ↑ oxidative stress - ↑ resorption rates	Folate deficiency reduces placental growth and development
Rosario et al. 2017 [107]	Mouse on folate-deficient diet before and during pregnancy	*Maternal folate deficiency:* - ↓ mTORC1 and mTORC2 signalling - ↓ trophoblast plasma membrane systems A and L amino acid transporter activities - ↓ trophoblast amino acid transporter isoform expression	Folate deficiency reduces amino acid transport to the fetus

Dark grey is table heading; pale grey titles demonstrate whether the study was clinical, ex-vivo or in vitro human placental, or in-vivo rodent studies. ⇔ no change; ↓reduction; ↑ increase. hCG, human chorionic gonadotropin; TNFα, tumour necrosis factor alpha; EGFR, epidermal growth factor receptor; TBH, *tert*-butylhydroperoxide; mTOR, mammalian target of rapamycin; DNMTs, DNA methyltransferase; ENG, endoglin; VEGF, vascular endothelial growth factor; PAPP-A, pregnancy-associated plasma protein A; hPL, human placental lactogen; ZEB2, zinc finger E-box binding homeobox 2; CDK6, cell division protein kinase 6.

**Table 3:** Rates of vitamin B_12_ deficiency in patients treated with metformin [57,58,59,111,131,132,133].

Reference	Study Description	Subjects	Definition of Serum B_12_ Deficiency	Rates of Vitamin B_12_ Deficiency	Dose of Metformin Associated with B_12_ Deficiency	Duration Associated with B_12_ Deficiency
Kim et al. 2019 [57]	Investigating B_12_ deficiency and >6 months of metformin treatment	1111 T2DM patients	≤300 pg/mL	Deficiency in 22.2% of patients, *n* = 247	>1000 mg/day	No association
Aroda et al. 2016 [58]	Investigating long-term effect of metformin use on vitamin B_12_ deficiency	1800 patients participating in the Diabetes Prevention Program (DPP)/DPP Outcomes Study (DPPOS)	≤203 pg/mL	4.3% at 1 year 19.1% at 5 years 20.3% at 13 years	Metformin 850 mg twice daily	1 year
Ahmed et al. 2016 [131]	Investigating the prevalance of vitamin B_12_ deficiency in T2DM patients treated with metformin	121 T2DM patients	<150 pmol/L	28.1%	2.4 ± 0.7 g/day	6 months
Beulens et al. 2015 [59]	Investigating B_12_ deficiency and metformin	550 T2DM patients	<148 pmol/L	Deficiency in 28.1% of patients	1 mg daily dose escalation = 0.042 pg/mL reduction in serum B_12_	No association
Ko et al. 2014 [132]	Investigating B_12_ deficiency and > 3 months of metformin treatment	799 T2DM patients	≤300 pg/mL	Deficiency in 9.5% of patients, *n* = 76	>1000 mg/d	>4 years
Gatford et al. 2013 [111]	Investigating vitamin B_12_ deficiency and metformin during pregnancy compared with insulin treatment	180 GDM patients: metformin (*n* = 89) vs. insulin (*n* = 91)	<148 pmol/L	No association	Treated with up to 2.5 g/day	No association
Tomkin et al. 1971 [133]	Assessment of vitamin B_12_ in patients taking long-term metformin therapy	71 patients with diabetes	<190 pg/mL	29.6% had vitamin B_12_ malabsorption	1.97 g/day	Not assessed

**Table 4:** Impact of metformin on folate, vitamin B_12_, and Hcy levels [58,69,112,140,141,142,143].

Reference	Subjects	Duration of Metformin Treatment	Dose of Metformin	Effect on Hcy, B_12_, and Folate
Esmaeilzadeh et al. 2017 [112]	18 females with PCOS	6 months	500 mg twice daily	Hcy ⇔ Serum folic acid ⇔ Serum vitamin B_12_ −20%
Aroda et al. 2016 [58]	1800 patients participating in the Diabetes Prevention Program (DPP)/DPP Outcomes Study (DPPOS) - 1217 female - 583 male	3.2 years plus an additional 9 years in selected cohort	850 mg twice daily	Vitamin B_12_: −10% at year 1; ⇔ at year 9 Hcy: + 5% at year 1; ⇔ at year 9
Malaguarnera et al. 2015 [69]	231 T2DM - 111 female - 120 male	8.2 ±4.6 years	Not documented	Plasma Hcy + 58.1% Plasma folic acid − 34.1% RBC folate − 37.6%
Sahin et al. 2007 [140]	165 T2DM - 99 female - 66 male	6 weeks	One to two tablets of 850 mg per day	Plasma Hcy + 19.6% Plasma folic acid − 11%
Pongchaidecha et al. 2004 [141]	152 T2DM	6 months	Not documented	Plasma Hcy ⇔ Serum folic acid ⇔ Serum vitamin B_12_ − 27%
Wulffele et al. 2003 [142]	353 T2DM - 186 female - 167 male	16 weeks	One to finally three tablets of 850 mg per day if tolerated	tHcy + 4% Serum folate − 7%
Carlsen et al. 1997 [143]	60 non-diabetic males with CVD	12 and 40 weeks	One group received up to 2000 mg metformin per day	tHcy: + 7.2% at 12 wks; + 13.8% at 40 wks Serum folate: ⇔ at 12 wks; − 8% at 40 wks

⇔ no change; ↓reduction; ↑ increase.

## Data Availability

No new data were created or analyzed in this study. Data sharing is not applicable to this article.
